# Pulmonary Embolism: Highlighting Iron Deficiency Anemia as a Contributing Factor in the Development of Pulmonary Embolism—A Case Report

**DOI:** 10.1002/ccr3.70158

**Published:** 2025-01-26

**Authors:** Fnu Yogeeta, Muskan Devi, Sameer Abdul Rauf, Fnu Tooba, Khadija Asif Sumar, Md Ariful Haque

**Affiliations:** ^1^ Liaquat National Medical College Karachi Pakistan; ^2^ United Medical and Dental College Karachi Pakistan; ^3^ Department of Public Health Atish Dipankar University of Science and Technology Dhaka Bangladesh; ^4^ Voice of Doctors Research School Dhaka Bangladesh; ^5^ Department of Orthopaedic Surgery Yan'an Hospital Affiliated to Kunming Medical University Kunming Yunnan China

**Keywords:** anticoagulant therapy, diagnostic challenges, iron deficiency anemia, pulmonary embolism, thrombosis

## Abstract

This case emphasizes iron deficiency anemia (IDA) as a potential risk factor for pulmonary embolism (PE), especially in patients with type 2 diabetes. Early recognition and management of PE and IDA are crucial. Further research is needed to clarify the mechanisms linking IDA to thrombosis and improve prevention strategies.

## 1 Introduction

The occurrence of pulmonary embolism (PE) ranges from 39 to 115 cases per 100,000 population, with individuals over 80 years of age being eight times more susceptible than those aged 40–50 [1, 2]. As people age, the incidence of PE tends to increase [3]. While men generally have a higher overall incidence of venous thromboembolism (VTE) compared to women (with a ratio of 1.2:1), women tend to experience a higher incidence after reaching the age of 75 [4]. PE can arise from various factors, including hematological disorders, contraceptive use, tumors, trauma, infection, and dehydration. Although infrequent, iron deficiency anemia (IDA) is recognized as a potential contributor to PE [5].

Low iron levels in the blood are often undermined as a risk factor for thromboembolism. Although secondary thrombocytosis associated with IDA is typically considered benign, emerging evidence suggests that elevated platelet counts, particularly in iron deficiency, can increase the risk of thromboembolism in both arterial and venous systems. Iron deficiency, a prevalent nutritional deficiency globally, is frequently associated with reactive thrombocytosis, which can lead to a hypercoagulable state [6]. This hypercoagulable state is one of the components of Virchow's triad, explaining the potential elevation of venous thromboembolism (VTE) risk. However, crucial but often overlooked risk factors such as IDA may not receive a thorough evaluation in routine assessments for VTE. Here, we share a case involving bilateral PE linked to IDA.

## 2 Case History/Examination

A 36‐year‐old woman with a known case of type 2 diabetes mellitus for the last 3 years presented in the emergency department with a complaint of sudden onset central chest pain, dull in character, radiating to the back of her shoulder, and dyspnea of grade 4 according to the modified MRC dyspnea scale, and sudden onset vomiting for 1 day. She also had a dry, nonproductive cough history for the last 3 days.

On general physical examination, her pulse was 126 bpm regular; her blood pressure was 120/80 mm/hg; respiratory rate was 28, tachypnea/min; oxygen saturation 75%, and cardiac examination showed S1 + S2 normal. Respiratory examination showed bilateral air entry reduced, CNS showed conscious and oriented, GI showed soft non‐tender, and hepatomegaly was found. Other examination showed dorsocervical fat pad, proximal myopathy, bruises, and striae reddish‐purple over the lower abdomen and thigh. The patient's BMI was 32.8 kg/m^2^, classifying her as Obese Class I.

## 3 Differential Diagnosis, Investigations and Treatment

**FIGURE 1:**
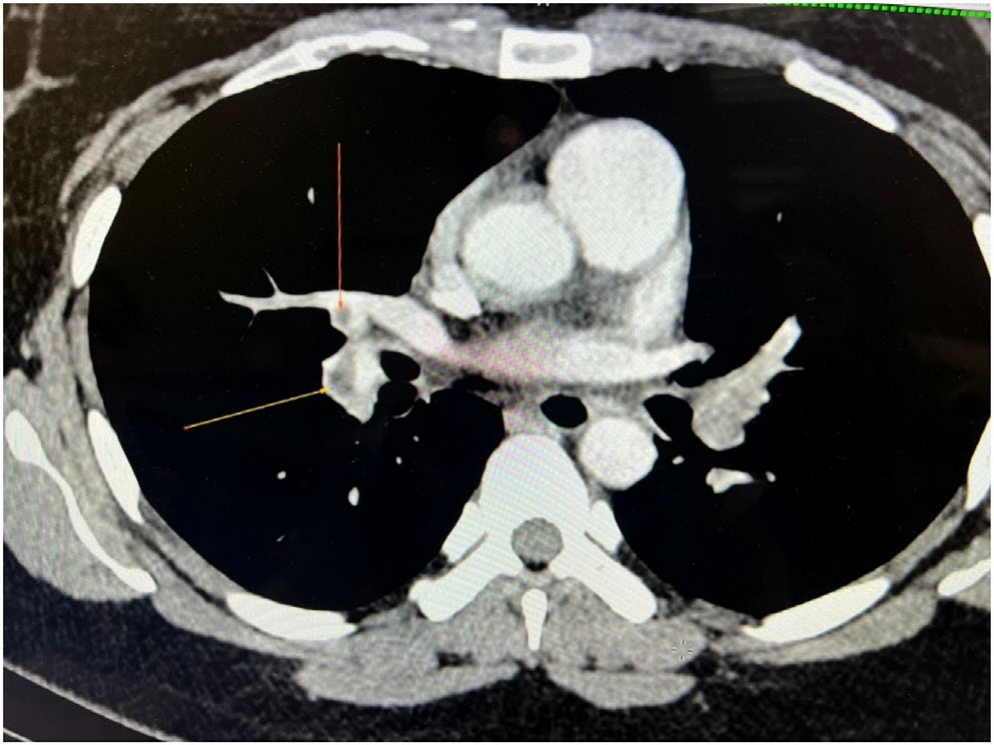
Shows a CT angiography image of the pulmonary arteries revealing an occlusion in the right main pulmonary artery.

**FIGURE 2:**
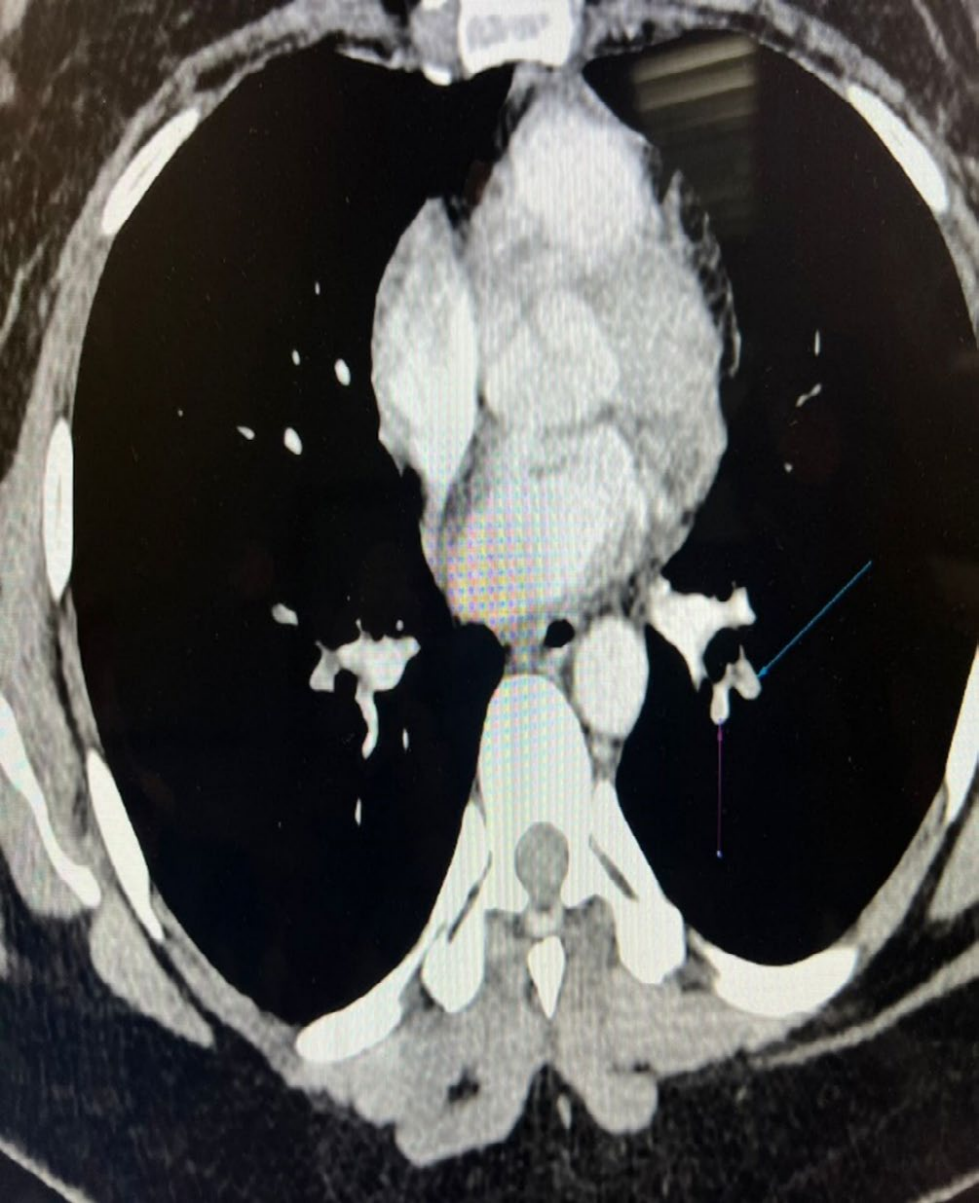
Shows a CT angiography of the pulmonary arteries, revealing a completely occluding hypodense thrombus in the left main pulmonary artery.

The results of the laboratory tests revealed significant findings (Tables 1 and 2). The CT angiography of pulmonary arteries showed a completely occluding hypodense thrombus in the left main pulmonary artery, extending into the upper and lower lobes of pulmonary arteries and their segmental branches. There was also a partial to nearly complete occluding hypodense thrombus in the distal right main pulmonary artery, extending into the lower lobe pulmonary artery and its segmental branches (Figures 1 and 2). On delayed images, peripheral and collateral filling of left lower lobe segmental branches were observed. Additionally, there was a hypodense filling defect in the right middle and right lower lobe pulmonary artery, extending into segmental branches with partial occlusion.

**TABLE 1:** Shows the results of several laboratory investigations.

Test	Results	Normal range
D‐dimer	1016.72 ng/mL	0–550 ng/mL
Procalcitonin	3.33 ng/mL (increased from 0.63 ng/mL)	—
Renal function test	Normal	—
Liver function test	Normal	—
Serum cortisol (9 am)	Normal	—
Serum cortisol (11 pm)	Slightly elevated: 18.27 μg/dL	—
Plasma ACTH	18.53 pg/mL	—
Free protein S assay	90.2%	50%–123%
Protein S assay	120.1%	72%–160%
Lupus anticoagulant	44.70s	36–50s
APLA IgG	2.39 U/mL	< 15 U/mL
APLA IgM	2.74 U/mL	< 15 U/mL
Homocysteine	15.95	4–15.3
HbA1C	8.0%	—
Cardio 3 panel	CKMB: 1.2 ng/mL	0.0–4.3 ng/mL
	Troponin I: 0.08 ng/mL	0.00–0.02 ng/mL
	BNP: 17.4 ng/mL	0.0–100 ng/mL
HBs‐Ag and HIV 1 and 2 rapid tests	Non‐reactive	—
Iron profile	Iron: 19.00 μg/dL	37–145 μg/dL
	UIBC: 502.40 μg/dL	110–370 μg/dL
	TIBC: 521.40 μg/dL	250–450 μg/dL
	Transferrin iron saturation: 3.64%	20%–50%
	Ferritin: 7.9 ng/mL	15–150 ng/mL
Urine Analysis	Turbid appearance	—
	Protein: 30+	—
	Glucose: 250	—
	Blood: 10+	—
C‐reactive protein (CRP)	125.1 mg/L	0–6 mg/L
ANA (indirect immunofluorescence assay)	Negative	—

**TABLE 2:** Shows the complete blood count (CBC).

Test	1st day	2nd day	3rd day	4th day
Hemoglobin (gm/dL)	8.3	9.0	10.0	10.3
Hematocrit (%)	26.2%	27.7%	30.3%	31.9%
MCV	56.0	59.0	57.8	58.8
MCH	17.7	19.2	19.1	19.0
Reticulocyte (%)	18.6%	21.1%	21.6%	21.6%
WBC (μL)	5.98 × 10^3^	7.40 × 10^3^	6.44 × 10^3^	9.37 × 10^3^
Platelets (μL)	414 × 10^3^	448 × 10^3^	504 × 10^3^	686 × 10^3^

**FIGURE 3:**
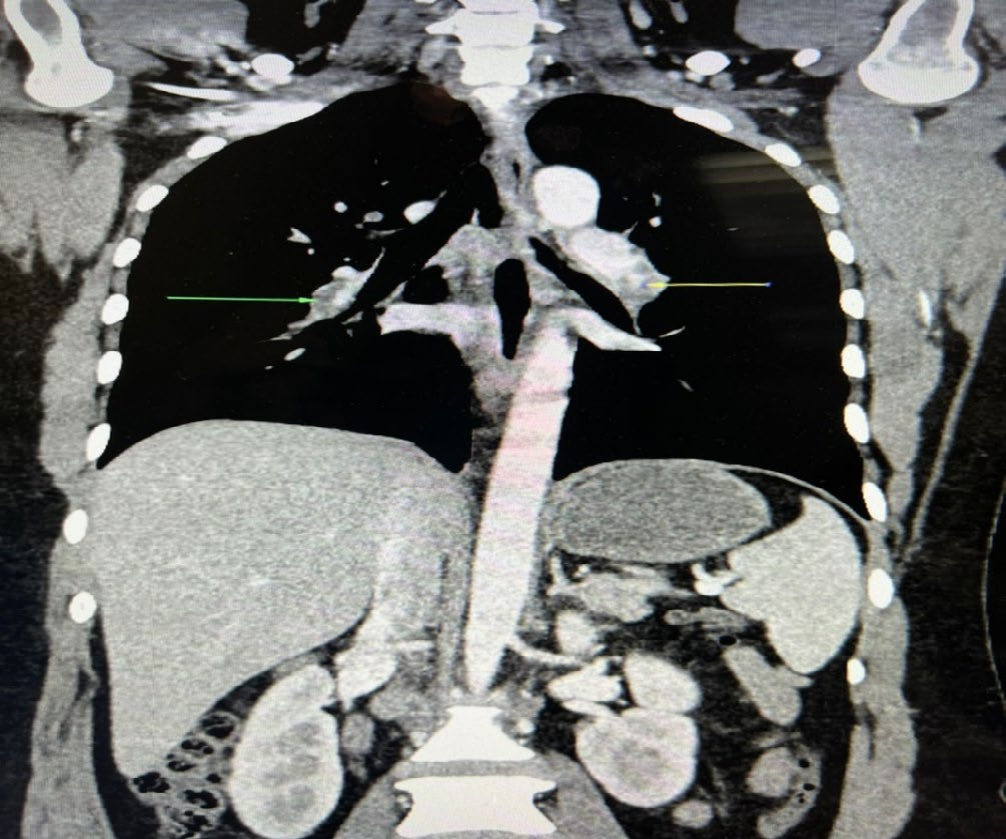
Shows a high‐resolution CT (HRCT) scan of the chest, revealing patchy areas of ground‐glass opacities in the right upper and middle lobes.

**FIGURE 4:**
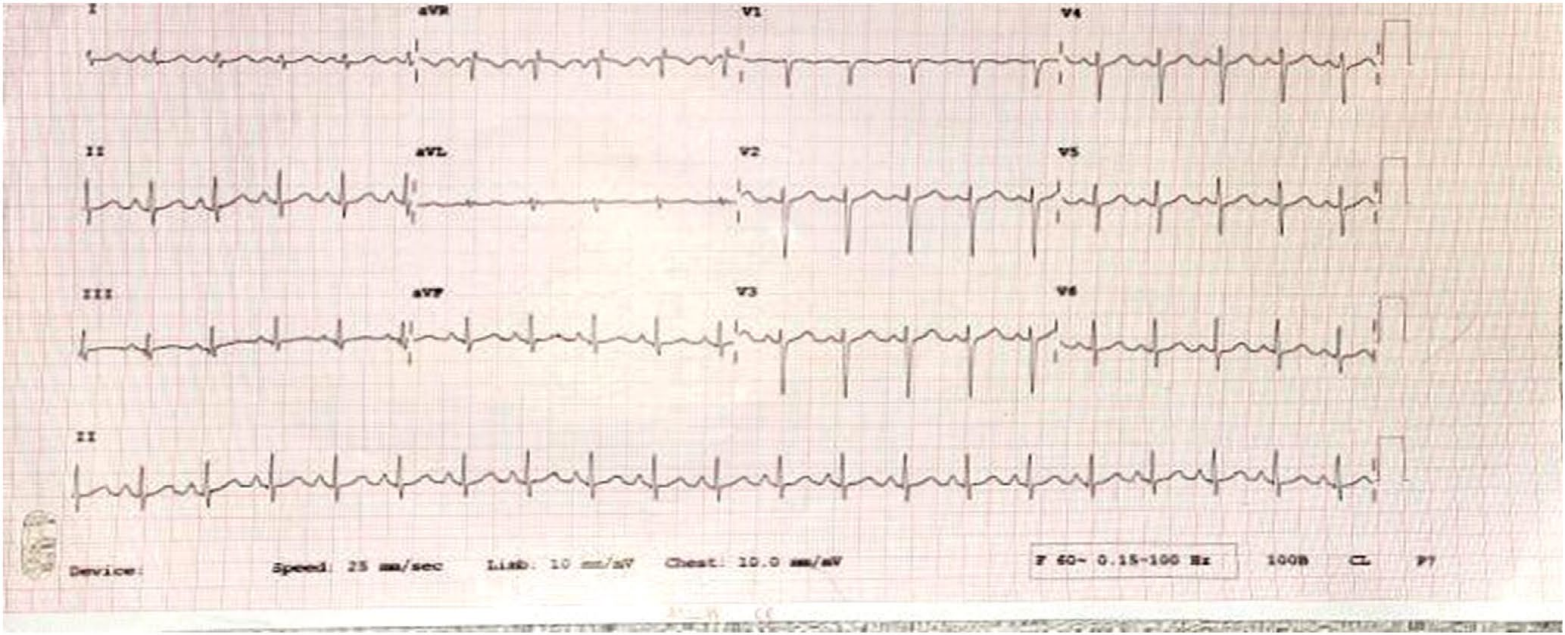
Shows an electrocardiogram (ECG) revealing sinus tachycardia.

On the other hand, the high‐resolution CT (HRCT) scan of the chest showed no active lung parenchymal lesions, consolidations, or ground glass opacities initially (Figure 3). However, on subsequent scans, patchy ground glass opacities were found in the right upper and middle lobes, suggesting an infective cause. Elevated inflammatory markers, including C‐reactive protein (CRP) at 125.1 mg/L (normal range: 0–6 mg/L) and procalcitonin at 3.33 ng/mL (normal range: < 0.5 ng/mL), indicated a systemic inflammatory response, consistent with an underlying infection that could contribute to thromboinflammation and the development of PE. Furthermore, on a later scan, areas of ground glass haziness were observed in the left lower lobe, and smooth septal thickening was seen in both lungs. Cardiomegaly was also noted, along with cholelithiasis, which is a calculus measuring approximately 8 mm. A 2D echocardiogram showed an ejection fraction of 55% and mild dilation of the right atrium and right ventricle with trace tricuspid regurgitation and mild mitral regurgitation. The estimated right ventricular systolic pressure (RVSP) was 40 mmHg, indicating mild pulmonary hypertension, consistent with the findings of PE. An electrocardiogram (ECG) showed sinus tachycardia (Figure 4). Wells' score for P.E. is 7.5 (high risk). The patient had no history of previous surgery, immobilization, use of estrogen therapy, or known hereditary thrombophilia, which could have served as additional predisposing factors.

Furthermore, laboratory tests and clinical evaluations indicated no underlying hemolysis. Lactate dehydrogenase (LDH) was 190 U/L (normal range: 120–250 U/L), and haptoglobin was 150 mg/dL (normal range: 30–200 mg/dL), both within normal limits and ruling out hemolysis as a contributing factor. The bilateral lower limb venous Doppler study revealed a normal venous scan without evidence of deep vein thrombosis (DVT).

Upon admission, the patient was given intravenous cefoperazone‐tazobactam (1.125 g) and pantoprazole (20 mg). When experiencing a sudden bout of vomiting, they received ondansetron (2 mL or 4 mg) intravenously until symptoms ceased. The prescribed medications also included rivaroxaban (15 mg) as an antithrombotic, rosuvastatin (40 mg) for lowering cholesterol, ivabradine (5 mg) as a heart medication, and pregabalin (40 mg) and insulin was administered with close monitoring of blood sugar levels. Upon discharge after 12 days, the patient received prescriptions such as cefuroxime with clavulanic acid, antibiotic eye drops, fluconazole (200 mg), ferrous ascorbate with folic acid, and a combination of domperidone and rabeprazole: Laxative, cough suppressant for symptomatic management. Diabetes treatment included insulin and gliptin; blood glucose levels were closely monitored.

After the initial five‐day regimen, treatment continued with Fluconazole 200 mg tablets and azithromycin 500 mg once daily for 5 days. Subsequently, the patient resumed ferrous ascorbate and folic acid tablets, rosuvastatin 40 mg tablets, rivaroxaban 15 mg tablets, insulin and oral diabetic medication, and steroids 6 mg tablets for ongoing care.

## 4 Conclusion and Results

This case underscores the importance of recognizing IDA as a potential risk factor PE, particularly in younger patients with comorbidities like diabetes mellitus. Prompt diagnosis and management, including anticoagulant therapy and iron supplementation, are crucial for optimal outcomes. Further research on the relationship between PE and IDA is needed, guiding improved risk assessment and tailored treatment strategies in clinical practice.

## 5 Discussion

PE is known as a challenging diagnosis due to its diverse and sometimes subtle symptoms, which can mimic those of various other medical conditions. Typical symptoms such as chest pain, Shortness of breath, pyrexia, cough, hemoptysis, and syncope often overlap with those of other clinical diagnoses. Typical symptoms and/or signs include chest pain, Shortness of Breath, pyrexia, cough, hemoptysis, and syncope. General physical examination may reveal increased heart rate, increased breathing rate, pyrexia, hypoxia, reduced breath sounds or rales, jugular venous distention, and right ventricular (RV) heave. General tests performed on these patients are 12‐lead electrocardiography (ECG) and/or chest X‐ray (CXR). The diagnosis of PE, likely stemming from IDA, was confirmed through chest CT angiography (CTA) and hematological analyses, ruling out other potential differential diagnoses related to PE.

Anticoagulant treatment is the best therapy for the treatment of acute PE. It should be given to all patients suspected of the disease and in the absence of active bleeding [7, 8]. Direct‐acting oral anticoagulants are preferred for treating PE except in pregnancy, severe renal impairment, or antiphospholipid syndrome, where vitamin K antagonists are preferred. Pregnant women and those with severe renal impairment are recommended to use low‐molecular‐weight heparin. Early discharge at 24 h is considered for patients without severe comorbidities or high risk of sudden death, provided proper home management and follow‐up can be ensured [9]. This advice is based on findings from the HESTIA trial, which explored treating low‐risk PE patients at home. Results showed only 2% experienced recurrent VTE, none within the initial 7 days of treatment [10]. Regular follow‐up is crucial for all PE patients due to higher cancer risk, potential bleeding complications, and the risk of chronic thromboembolic pulmonary hypertension, which may not be initially detectable [9].

Despite knowing that IDA is linked to blood clotting, we still lack much information about how significant this connection is, where in the body the clots tend to form, and which groups of patients are most at risk. Clinical studies have not fully explained how factors like low iron levels, anemia, or increased platelet count due to iron deficiency contribute to the higher risk of blood clots in people with IDA. Additionally, different studies use different criteria and levels to diagnose iron deficiency or anemia, making it hard to compare findings across research. This lack of standardized diagnostic methods and understanding of the underlying reasons highlights the need for more research. We need to understand better how IDA and blood clots are connected and figure out which patients are most likely to have problems so we can develop targeted ways to prevent blood clots in those groups [11].

Numerous investigations have documented a range of venous thromboembolism (VTE) occurrences in patients with IDA [12, 13, 14]. In comparison to individuals with IDA and no thrombocytosis, Song et al. calculated two times the increased thrombotic risk associated with reactive thrombocytosis in patients with IDA using large‐volume clinical data from an institutional research database that included 36,327 people with IDA [15]. However, a retrospective study disputes the connection between PE and IDA [16]. Uncertainties persist regarding the mechanisms underlying thrombocytosis and VTE in IDA.

Several theories have attempted to explain this link. Firstly, thrombopoiesis, the process by which platelets are produced, is significantly regulated by iron [17]. Adequate iron levels are necessary to suppress thrombopoiesis and prevent thrombocytosis. Consequently, IDA is linked to the absence of thrombocytosis inhibition, thereby increasing the risk of thrombosis [13]. Moreover, alternative pathogenic processes have been suggested because not all iron‐related thrombotic events occur in patients with a high platelet count.

Moreover, one proposed mechanism revolves around the role of iron as an antioxidant. Some authors have advised that reducing antioxidant defense in iron‐deficient anemia may lead to increased oxidative stress, predisposing to platelet aggregation and the increased thrombotic risk associated with thrombocytosis [18]. Another theory involves the altered blood flow pattern associated with iron shortage. Iron‐deficient red blood cells are more viscous and less deformable, potentially altering blood flow patterns inside vessels and leading to a hypercoagulable state [19]. Additionally, hypoxia due to IDA may manifest as the oxygen‐carrying capacity of red blood cells diminishes, particularly in scenarios where metabolic demands are heightened. These factors lead to disturbed blood flow patterns, increasing platelets' chances of contact with the endothelial lining more frequently [19].

## Author Contributions

**Fnu Yogeeta:** conceptualization, data curation, formal analysis, investigation, methodology, resources, software, writing – original draft. **Muskan Devi:** data curation, formal analysis, investigation, methodology, resources, software, validation, writing – original draft. **Sameer Abdul Rauf:** data curation, formal analysis, investigation, methodology, resources, software, validation, writing – review and editing. **Fnu Tooba:** data curation, investigation, methodology, resources, software, validation, visualization, writing – review and editing. **Khadija Asif Sumar:** data curation, formal analysis, investigation, methodology, software, validation, visualization, writing – review and editing. **Md Ariful Haque:** data curation, investigation, methodology, project administration, supervision, validation, visualization, writing – review and editing. All authors approved the final version to be published.

## Ethics Statement

Ethical approval was obtained from Liaquat National Hospital and Medical College.

## Consent

Written informed consent was obtained from the patient for publication of this case report and any accompanying images. A copy of written consent is available for review by the Editor‐in‐Chief of this Journal.

## Conflicts of Interest

The authors declare no conflicts of interest.

## Data Availability

The authors have nothing to report.
